# Distributed radiomics as a signature validation study using the Personal Health Train infrastructure

**DOI:** 10.1038/s41597-019-0241-0

**Published:** 2019-10-22

**Authors:** Zhenwei Shi, Ivan Zhovannik, Alberto Traverso, Frank J. W. M. Dankers, Timo M. Deist, Petros Kalendralis, René Monshouwer, Johan Bussink, Rianne Fijten, Hugo J. W. L. Aerts, Andre Dekker, Leonard Wee

**Affiliations:** 1 Department of Radiation Oncology (MAASTRO), GROW School for Oncology and Developmental Biology, Maastricht University Medical Centre+, Maastricht, The Netherlands; 2 Department of Radiation Oncology, Radboud University Medical Center, Nijmegen, The Netherlands; 3 The D-Lab: Decision Support for Precision Medicine, GROW-School for Oncology and Developmental Biology, Maastricht University Medical Center+, Maastricht, The Netherlands; 4 Department of Radiation Oncology & Radiology, Dana-Farber Cancer Institute, Brigham and Women’s Hospital, Harvard Medical School, Boston, MA United States of America; 5 Radiology and Nuclear Medicine, Maastricht University Medical Center+, Maastricht, The Netherlands; 6 Radiation Medicine Program, Princess Margaret Cancer Centre, Toronto, Canada

**Keywords:** Radiography, Machine learning, Computational models, Non-small-cell lung cancer

## Abstract

Prediction modelling with radiomics is a rapidly developing research topic that requires access to vast amounts of imaging data. Methods that work on decentralized data are urgently needed, because of concerns about patient privacy. Previously published computed tomography medical image sets with gross tumour volume (GTV) outlines for non-small cell lung cancer have been updated with extended follow-up. In a previous study, these were referred to as Lung1 (n = 421) and Lung2 (n = 221). The Lung1 dataset is made publicly accessible via The Cancer Imaging Archive (TCIA; https://www.cancerimagingarchive.net). We performed a decentralized multi-centre study to develop a radiomic signature (hereafter “ZS2019”) in one institution and validated the performance in an independent institution, without the need for data exchange and compared this to an analysis where all data was centralized. The performance of ZS2019 for 2-year overall survival validated in distributed radiomics was not statistically different from the centralized validation (AUC 0.61 vs 0.61; p = 0.52). Although slightly different in terms of data and methods, no statistically significant difference in performance was observed between the new signature and previous work (c-index 0.58 vs 0.65; p = 0.37). Our objective was not the development of a new signature with the best performance, but to suggest an approach for distributed radiomics. Therefore, we used a similar method as an earlier study. We foresee that the Lung1 dataset can be further re-used for testing radiomic models and investigating feature reproducibility.

## Introduction

Images from radiological examinations are presently one of the largest underutilized resources in healthcare “big data”^1^. *Radiomics* refers to computerized extraction of quantitative image metrics, known as “features”. In 2014, Aerts *et al*.^2^ showed that radiological features from Computed Tomography (CT) scans might encode additional information about phenotypic differences between tumours that lie beyond the grasp of the unaided human eye. The hypothesis is that multifactorial prediction models incorporating selected radiomic features may better inform individually personalized treatment strategies^3–6^. Radiomic data have now been investigated in CT^7–9^, magnetic resonance imaging (MRI)^10,11^ and positron emission tomography (PET)^12,13^.

The availability of commercial and open source software for radiomic feature extraction has made this line of inquiry accessible to a large number of investigators^14–17^. However, multi-institutional development and validation of radiomic-assisted prediction models is slowed down due to privacy concerns about sharing of individual patients’ medical images. Significant efforts are under way to make image sets used in radiomic investigations openly accessible via centralized repositories such as The Cancer Imaging Archive (TCIA; https://www.cancerimagingarchive.net)^18^, however, many data owners remain cautious about sharing individual patient images publicly online.

A privacy-preserving distributed learning infrastructure based on World Wide Web Consortium “Semantic Web” data sharing standards^19^, known as Personal Health Train (PHT; https://vimeo.com/143245835)^20^ has been successfully used to develop and validate models on non-image clinical data^21–23^. To extend the PHT approach to radiomics, we first need to publish our radiomic features in a manner that is Findable, Accessible, Interoperable and Re-useable (FAIR)^24^. We have developed a pragmatic and extensible Radiomics Ontology (RO) that is publicly accessible via NCBO BioPortal (https://bioportal.bioontology.org/ontologies/RO). With the RO, we can describe over 430 class objects and 60 predicates between objects to publish radiomic features (with some relationships and dependencies) according to Semantic Web standards. The class objects include unique feature identifiers that are aligned with the Image Biomarker Standardization Initiative (IBSI)^25^.

In this article, we show that the PHT infrastructure supports exchange of cross-institutional radiomic-based clinical data without material transfer of individual-level patient clinical data or images. Our primary objective was to show that external validation of a radiomic signature can be done with entirely decentralized data.

The specific use case was to learn a radiomic signature “ZS2019” for non-small cell lung cancer (NSCLC) overall survival at one institution and validate it at a remote institution in a distributed fashion. We included two of the NSCLC subject cohorts used by Aerts *et al*.^2^, however, with independently reviewed annotations (tumour delineations) and extended follow-up times for overall survival. We did not select new radiomic features, and instead used the four features corresponding to those described previously in the original publication, but using a different software implementation (see materials and methods). The first of these datasets (hereafter referred to as “Lung1”)^26^ was generated at Maastricht University, which was used exclusively for model training, thus obtaining coefficients for a four-feature signature in ZS2019. The second of these datasets (hereafter “Lung2”) was generated at Radboud University remains in a private hospital collection that could not be shared publicly for privacy reasons; Lung2 was used exclusively for model validation.

## Results

Cohort summary information was exchanged through private discussion between the collaborating investigators, prior to performing this study. This was to confirm that general characteristics were comparable between the updated cohorts. This is shown in Table 1. None of the information contained in Table 1 was used in the model. There was a slightly higher proportion of patients with metastatic disease in Lung2 (10% vs 1%) compared to Lung1. The most common histology types in Lung1 were large-cell and squamous-cell carcinomas, whereas adenocarcinoma and squamous-cell carcinoma were most common in Lung2. The median follow-up time, the median survival time and the overall 2-year survival rate were similar in both cohorts.

**Table 1:** The clinical case-comparison for the training cohort (Lung1) and the validation cohort (Lung2). The abbreviations are: (GTV) is Gross Tumour Volume delineated on the radiotherapy treatment planning computed tomography image, (Clinical T) is the tumour staging, (Clinical N) is the node staging and (Clinical M) is the metastasis staging, respectively, according to the TNM tumour classification system.

	Lung1 (n = 421)	Lung2 (n = 221)
Median age (range) at diagnosis in years	68.5 (34–92)	66.0 (36–87)
Median GTV size (range) in cm^3^	39 (0–660)	88 (1–860)
Clinical T stage *Less than 3* *3 or greater* *Unknown*	249 (59%) 171 (41%) 1 (0%)	119 (54%) 85 (38%) 17 (8%)
Clinical N stage *0* *1* *2 or greater* *Unknown*	170 (40%) 22 (5%) 229 (55%) 0 (0%)	49 (22%) 16 (7%) 137 (62%) 19 (9%)
Clinical M stage *0* *1 or greater*	416 (99%) 5 (1%)	200 (90%) 21 (10%)
Histology *Adenocarcinoma* *Large-cell* *Squamous cell carcinoma* *Other*, *or not otherwise specified* *Unknown*	51 (12%) 143 (34%) 152 (36%) 63 (15%) 12 (3%)	64 (29%) 22 (10%) 82 (37%) 47 (21%) 6 (3%)
Outcomes *Median follow-up in days* *Median survival time in days* *2-year overall survival rate*	546 478 40%	595 500 41%

We evaluated ZS2019 for 2-year overall survival using multivariable logistic regression. The area under the receiver operating characteristic curve (AUC) discrimination metric was 0.61 (95% confidence interval: 0.54 to 0.69) in the Lung2 validation cohort.

**Fig. 1:**
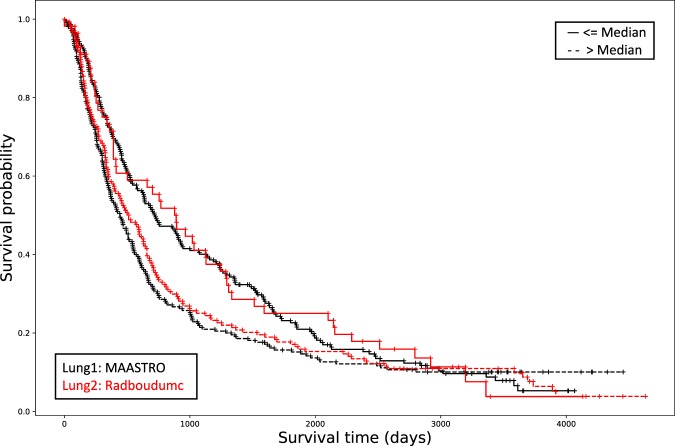
The performance of radiomic signature ZS2019 according to Kaplan-Meier survival analysis. The signature was developed in Lung1 (MAASTRO; black line) and then distributedly validated in Lung2 (Radboudumc; red line). The upper and lower survival curves were split according to the median of the Cox regression linear predictor from the Lung1 data, and applied to both Lung1 and Lung2 data. The Harrell concordance index in the test cohort was 0.58, the log-rank test yielded a p-value of 0.09 and the Wilcoxon test gave p-value < 0.0001.

Distributed learning code for Cox regression in MATLAB (MATLAB 2016a, Mathworks, Natick MA, USA) was deployed via the PHT infrastructure connecting MAASTRO Clinic and Radboudumc. We retrieved anonymous event timepoints and thus compiled Kaplan-Meier curves for overall survival in each of the training and validation cohorts (in Fig. 1). Within each cohort, the subjects were stratified into two risk groups, based on the median of the risk score distribution in Lung1. Stratification of survival curves by ZS2019 in the validation cohort was quantified via a Harrell Concordance Index (HCI) of 0.58, and a 95% confidence interval from 0.51 to 0.65. The discrimination was statistically significantly different from random (p < 0.0001) based on a bootstrapped Wilcoxon estimation. We performed the same bootstrapped Wilcoxon estimation between the mean HCI of model ZS2019 (0.58) and the HCI previously published by Aerts *et al*. (0.65)^2^, and found no evidence of significant divergence (p = 0.37).

We confirmed that the same ZS2019 result was obtained when trained centrally on Lung1 and validated in Lung2. The analysis is given in a Python v3.6 JuPyter notebook that is made publicly available (https://gitlab.com/UM-CDS/distributedradiomics). The central data approach yielded a HCI of 0.58 with a 95% confidence interval estimated by bootstrap sampling to be 0.53 to 0.64.

## Discussion

In this paper, a model (ZS2019) derived from radiomic features and overall survival locally within one institution was able to be exchanged interoperably with an external institution, without mandating any transfer of either images, feature values or clinical outcomes at the individual subject level. This is an essential and unique contribution to radiomic investigations, because we hereby demonstrate the concept for carrying out multi-centre radiomic studies with fully decentralized data. The results obtained with decentralized data were the same as if all the data had been brought into the same location. However, the unique advantage of our approach is that no one party needs to risk breaking patient confidentiality by exposing the original data to another party. Each institutional data owner retains complete control over their privacy-sensitive patient data, and decides what they wish to share for a collaborative project.

We foresee that public access to the updated Lung1 dataset, accessible together with open source radiomics software code, encourages re-use of the data for validating models, investigating radiomic feature generalizability and deep-learning for image analysis.

To learn effectively across institutions, it is essential that the investigation should be led by clinical experts. Our approach does not bypass the need for human experts to communicate extensively before commencing a study, in order to establish consensus on: (i) what is the clinical question to be addressed, (ii) relevant inclusion and exclusion criteria, (iii) which datasets are appropriate for answering the question and (iv) how to define the radiomic features and outcome concepts.

With respect to handling errors and discrepancies for a distributed radiomics study, it is essential that each data owner takes responsibility for curation and quality assurance of the data, such that it conforms to the agreed consensus. Where errors are detected, it is only the owners of the data that are able to review, contextualize and correct their own data.

 In this study, both sites used the same feature extraction software, PyRadiomics. We retained the step of attaching metadata to the features using the Radiomics Ontology so that, in future, sites might be able to use different software but can still understand each other because features having the same metadata labels from this ontology will be unambiguously defined as being semantically identical. Besides applying an ontology, this also requires the different Radiomics feature extraction software to use the (exact) same feature calculation method.

The approach of making data FAIR using semantic ontologies has the benefit of allowing each data owner to keep their own native language and annotation conventions in the original data. No syntactic harmonization of the data below the level of the FAIR station needs to be enforced, and no data code-books need to be exchanged. The only prerequisite here is that partnering institutions must follow their consensus agreement to label the comparable outcomes and equivalent radiomic features with the same unique identifier from the same domain ontology.

To develop ZS2019, we attempted to follow, as closely as possible, the approach adopted in the original publication. The HCI and AUC results we reported above were built using radiomic features that might not be optimal for the updated datasets, because we chose to use the four features with names corresponding to those described previously in the supplementary material of the prior study^27^. Development of an optimal radiomic signature for NSCLC overall survival would require a detailed re-examination of features and feature selection in the updated datasets, which is not the primary objective of the present study.

The PHT approach utilises existing data to answer key questions in personalised healthcare, preventive medicine and value-based healthcare. PHT is one of a number of innovative approaches (DataSHIELD^28^ and WebDISCO^29^) where the research question is coded as machine-learning algorithms sent to wherever data may reside, instead of centralising all of the data at one location. This is achieved by (i) creating FAIR data stations, (ii) creating “trains” containing the research question as a machine-learning algorithm and (iii) establishing “tracks” to regulate the trains and securely transmit them to data stations. The PHT is thus a “privacy-by-design” architecture, since it enables controlled access to heterogeneous data sources for clinical research. This respects data protection and personal privacy regulations, and requires active engagement of data owners in the process.

We used Semantic Web standards to make radiomic features and outcome data available as FAIR stations in keeping with our trains metaphor. This included locally storing radiomic features and outcome states in Resource Description Format (RDF), and allowing semantic interoperability using a combination of the Radiomics Ontology and Radiation Oncology Ontology. The benefit of Semantic Web is to make distributed learning possible even if the underlying implementation of data extraction and storage differs between sites. The RDF standard makes it unnecessary to first know the internal structural organization of a remote database in order to successfully execute a local data retrieval query. Furthermore, as the diversity and complexity of the data within the FAIR stations increases in the future, an RDF triple store approach is sufficiently flexible to describe arbitrarily complex concepts without the need to redesign the database.

 Use of the Varian Learning Portal (VLP; Varian Medical Systems, Palo Alto, USA) was of benefit for distributed radiomics, because the software had already implemented the essential technical overheads (logging, messaging and internet security) required for such distributed studies. This included underlying legal agreements between the parties and Varian, that makes distributed radiomics more scalable since one does need to revisit these common aspects above for each project. The VLP system had no effect on the mathematical results of our study because it was purely a way for us to securely transmit learning algorithms and trained models. Alternatives to VLP such as DataSHIELD (http://www.datashield.ac.uk)^28^, WebDisco (https://omictools.com/webdisco-tool)^29^ and ppDLI (https://distributedlearning.ai/blog) may also be used for distributed radiomics. The differences between the present study and the original study may be traced to: (i) the original Matlab code is commercial confidential and not available to the authors, so we used PyRadiomics developed by van Griethuysen *et al*.^15^ as a practical alternative and (ii) we tried our best to replicate the original method using the documented steps in the original manuscript, but we also improved the survival follow-up such that many right-censored events were now confirmed deaths.

## Conclusion

This study demonstrates the proof of concept for multi-centre distributed radiomics investigation without exchanging individual-level data or medical images using the PHT infrastructure. The results showed that the proposed decentralized approach achieved the identical results as the fully centralized approach. Moreover, we performed a radiomics study where data was stored in the FAIR station at the institute rather than publishing as open-source. Finally, the work of this study may be used as the basis for other types of radiomics studies such as binary classification or regression, not only limiting to survival analysis.

## Methods

### Patients

Subjects in this replication study were from the same cohorts of non-small cell lung cancer (NSCLC) patients previously treated with (chemo-)radiotherapy at MAASTRO Clinic (MAASTRO) and Radboud University Medical Centre (Radboudumc). These were previously labelled by Aerts *et al*.^2^ as cohorts “Lung1” and “Lung2”, respectively, and the same nomenclature is followed in this study. The Lung1 cohort (n = 421) was used only for fitting of model coefficients, and Lung2 (n = 221) was exclusively used for external validation.

### Tumour delineations

Radiotherapy treatment planning DICOM CT images and physician-delineated primary NSCLC tumours as RT structure sets were used. From 422 available, 34 cases were found to have a reference frame translation between the image and delineation due to incorrect coding of the treatment couch height offset in the planning system; these have been rectified for the TCIA collection. Only 1 patient was post-operative radiotherapy, so this case was excluded from any further analysis, leading to 421 eligible cases in Lung1 for model training.

In the Lung2 cohort, there were initially 267 subjects available. A check against delineation criteria found 221 eligible primary tumours for radiomic analysis. The other 46 patients had either gross tumour volumes including lymph nodes, or were cases with neoadjuvant treatment or had no primary tumour in the list of structures.

### Outcomes

Updated follow-up intervals in early 2018 with recent dates of death were obtained with ethics board permission from the Dutch citizens registry. As expected, the number of registered deaths in Lung1 and Lung2 had increased significantly since the original publication. The time intervals from date of first radiotherapy fraction to date of either registered death or last known survival were updated in both Lung1 and Lung2.

### Data processing

**Fig. 2:**
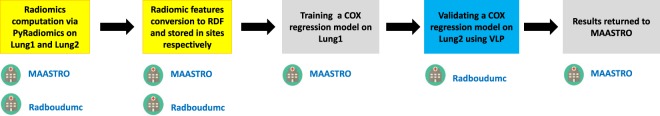
A schematic diagram explaining the primary methodology for survival analysis used in this study. Details have been provided in the text. Briefly, radiomics features were extracted locally by each institution and then labelled with the radiomics ontology. We then trained a Cox regression model on Lung1 (MAASTRO) and then validated on Lung2 (Radboudumc) by distributing the learning algorithm through the Varian Learning Portal (VLP). Only the event coordinates required to plot a Kaplan-Meier survival curve was returned to MAASTRO, without any identifiable patient-level data.

The study steps are shown schematically in Fig. 2 for MAASTRO and Radboudumc. The core of the radiomic feature extraction process utilizes free and open-source PyRadiomics^15^ (v1.3) libraries. Software wrapper extensions collectively known as O-RAW (https://gitlab.com/UM-CDS/o-raw) were used to convert DICOM objects into numerical arrays as inputs for PyRadiomics; these were based on the SimpleITK (v1.0.1)^30^ toolkit.

The original MATLAB scripts used by Aerts *et al*. were not accessible to the current authors. The open source PyRadiomics was developed independently of this MATLAB code, and was based on the original study from Aerts *et al*. The PyRadiomics community has documented and standardized the feature calculation formulae (https://pyradiomics.readthedocs.io).

The image pre-processing methodology was the same as in the original publication^2^; an extraction intensity bin width was set at 25 Hounsfield Units with no image resampling and no image intensity normalization. The coif1 wavelet package from the pywavelets library (v0.5.2, https://github.com/PyWavelets/pywt) was used to generate wavelet features with a starting bin edge of 0. All of these settings are the default in PyRadiomics.

For the development of ZS2019 we did not select new radiomic features, and instead used the four features with names corresponding to those described previously in the supplementary material^27^ that accompanied the original publication:

i. Energy from the intensity histogram feature class, which estimates the overall density of the region of interest,
ii. Compactness from the morphological feature class, which describes the volume of the object relative to that of a perfect sphere,
iii. Grey level run-length matrix (GLRLM) non-uniformity from the textural feature class, which is a measure of intensity heterogeneity averaged over 13 different directions in a 3D matrix of values, and
iv. Wavelet-filtered (HLH) GLRLM non-uniformity, which was the same as (iii) after applying a wavelet decomposition filter over the original image.

In our work, the feature “compactness” had been deprecated in PyRadiomics, so we derived the mathematical equivalent of compactness by taking the cube of the shape feature “sphericity” (see formulae in Table A of Supplementary Materials).

### Semantic web ontologies

Semantic Web technologies and ontologies play a key role in distributed learning by enabling semantic interoperability between data from multi-centres. In this study, radiomic features and clinical data were defined by a Radiomics Ontology v1.3 (https://bioportal.bioontology.org/ontologies/RO) and a Radiation Oncology Ontology^31^, respectively.

We elected to use the published open access Radiomics Ontology, that identifies radiomic features via a globally persistent unique identifier and allows us to attach important dependencies, such as digital image pre-processing steps, directly to each given feature. Though radiomic features definitions have been defined by previous investigators, our contention is that human-readable labels alone may not always be easily extensible to define dependencies such as software versions, image pre-processing steps and mathematical implementation of the feature. For example, to avoid conflation between features labelled “entropy”, the IBSI distinguishes between Intensity Histogram Entropy (unique ID = TLU2) and the textural feature Joint Entropy (unique ID = TU9B). The Radiomic Ontology allows extensible and adaptable declaration of radiomic feature provenance by publishing it as a data graph object. Therefore, independent researchers (in the aforementioned example) who have computed Joint Entropy may use the SPARQL federated query language (https://www.w3.org/TR/rdf-sparql-query) on feature graphs to also probe for similarities in imaging setting, pre-processing methods, and suchlike. We hypothesise that the data graph based approach is more scalable than pairwise cross-referencing of multiple dictionaries of feature definitions.

### Distributed approach

The VLP distributed learning architecture has been described in deep detail elsewhere^21–23^. In brief, VLP consists of (i) a global web-based clinical learning environment that spans across any number of participating institutes for a given learning project, and (ii) a local connector application that runs exclusively inside the IT firewall of each institute. The former coordinates access permission, asynchronous messaging, web security and site privacy protocols across the learning network, while the latter hosts a local FAIR data repository. Radiomic feature values were hosted in the respective VLP local connector application (v2.0.1) as RDF.

Authenticated and verified (e.g. encrypted digital signature) machine learning packages are distributed via the global part of VLP, then picked up and executed on the RDF data via the local connector part. Only the statistical summary result of the computation, not any identifiable patient data, is thereafter passed back to the instigator via the global VLP part. Any process that had executed within local firewalls remain permanently quarantined from the global part.

### Model training

The Lung1 radiomic feature values were log-transformed and then scaled to z-scores. A multivariable Cox proportional hazards model for overall survival (with removal of right censored subjects not yet deceased) was then fitted using all of the available subjects in the training cohort. The median risk score in the training cohort was recorded and thus used to stratify the training population into two risk groups. The fitted Cox model coefficients, the median risk score and the z-score transformations from the training cohort were packaged as self-contained validation application, which was then transmitted via VLP to Radboudumc.

At Radboudumc, the application queried the local RDF repository for the radiomic features, then applied the same log-transform of raw feature values and the same z-score scaling as had been executed on Lung1. For each available validation subject in Lung2, the risk score was computed and stratified according to the median risk score of Lung1. A flat table of individual timepoints and death/censor events was sent back via VLP to MAASTRO.

### Cox model evaluation

Anonymous timepoints for Kaplan-Meier survival curves^32^ were retrieved over the PHT infrastructure. Risk scores were stratified into two strata according to the median value in the Lung1 population. A Harrell concordance index (HCI)^33^ implemented using the python lifelines package (v0.14.4) was used to quantify discrimination performance using the retrieved timepoints. The log-rank method^34^ was used to calculate a chi-squared test statistic and p-value for the significance of the discrimination. To assess if the survival model had any value beyond random discrimination (null hypothesis: c-index = 0.5), we used a two-sided Wilcoxon test with a bootstrap approach on 100 repeated sub-samples of 100 patients per repetition from Lung2.

### 2-year overall survival

A multivariable logistic regression model for 2-year overall survival was developed on Lung1 then validated on Lung2 using the aforementioned four features. The area under the curve of the receiver operating characteristic was used to assess the discrimination. The bootstrap method (1000 times) was used to estimate a 95% confidence interval around the mean AUC.

## Supplementary information

Supplementary Materials.

## Data Availability

The Lung1 images, primary tumour delineations (from Method: tumour delineations) and clinical outcomes with updated follow-up (from Method: outcomes) has been approved for open access publication, and is curated as the collection called “NSCLC-Radiomics” via The Cancer Imaging Archive (TCIA)^26^. The clinical data for Lung1 that support the findings of this study are also available in TCIA with the data identifier (10.7937/K9/TCIA.2015.PF0M9REI). Further information regarding the Lung1 data may be obtained from the authors responsible, A Dekker (email: andre.dekker@maastro.nl; address: Doctor Tanslaan 12, 6229 ET; Maastricht, The Netherlands; phone: +31 88 445 5600) and L Wee (email: leonard.wee@maastro.nl; address: Doctor Tanslaan 12, 6229 ET; Maastricht, The Netherlands; phone: +31 88 445 5600). The Lung2 dataset that support the findings of this study are available by request from the authors R Monshouwer (email: rene.monshouwer@radboudumc.nl; address: Radboud university medical center, Department of Radiation Oncology, Geert Grooteplein 32, 6525 GA, Nijmegen, The Netherlands; phone: +31 24 361 4515) and J Bussink (email: jan.bussink@radboudumc.nl; address: Radboud university medical center, Department of Radiation Oncology, Geert Grooteplein 32, 6525 GA, Nijmegen, The Netherlands; phone: +31 24 361 4515). This part of data are not publicly available due to the data containing information that could compromise research participant privacy.

## Code availability

The code used in this study is made publicly available on the Maastricht University Clinical Data Science (UM-CDS) GitLab repository (https://gitlab.com/UM-CDS/distributedradiomics). The code repository has the following organization:

a. D2RQ folder: contains the raw feature value to RDF mapping (D2RQ) script and the SPARQL query used to retrieve the local data into the local VLP connector application.

b. VLP folder: contains the MATLAB codes submitted by the user into VLP, which then transmits it to the participating site for model validation and analysis.

c. Analysis Centralized Learning folder: contains the Jupyter notebook from Radboudumc for model development and evaluation on the aggregated datasets.

The open-access Radiomics Ontology (RO) is published via the National Center for Biomedical Ontology (NCBO) ontology registry. It is available to download in a range of formats from the following URL: https://bioportal.bioontology.org/ontologies/RO. As a domain ontology, the RO defines histogram-based, morphology-based and texture-based radiomic features, including (since v.1.6, 08 November 2018) all feature entities presented in the International Biomarker Standardization Initiative. The ontology also defines software properties, digital imaging filter operations and feature extraction settings, together with relational predicates to link these to each feature entity.
