# Supplementary material for: Distributed radiomics as a signature validation study using the Personal Health Train infrastructure
Source: Sci Data. 2019 Oct 22;6:218. doi: 10.1038/s41597-019-0241-0 (PMC6805885; doi:10.1038/s41597-019-0241-0)
Supplement: Supplementary file 1 — Supplementary Materials. [file 41597_2019_241_MOESM1_ESM.docx]

**Supplementary**

### Distributed Radiomics as a signature validation study using the Personal Health Train infrastructure

### Authors

Zhenwei Shi**^*,†,^**^1^

Ivan Zhovannik**^†,1,,^**^2^

Alberto Traverso^1,6^

Frank J.W.M. Dankers^1,2^

Timo M. Deist^1,3^

Petros Kalendralis^1^

René Monshouwer^2^

Johan Bussink^2^

Rianne Fijten^1^

Hugo JWL Aerts^4,5^

Andre Dekker^1^

Leonard Wee^1^

**Affiliations**

1. Department of Radiation Oncology (MAASTRO), GROW School for Oncology and Developmental Biology, Maastricht University Medical Centre+, Maastricht, The Netherlands

2. Department of Radiation Oncology, Radboud University Medical Center, Nijmegen, The Netherlands

3. The D-Lab: Decision Support for Precision Medicine, GROW-School for Oncology and Developmental Biology, Maastricht University Medical Center+, Universiteitssingel 40, 6229 ER, Maastricht, The Netherlands

4. Department of Radiation Oncology & Radiology, Dana-Farber Cancer Institute, Brigham and Women’s Hospital, Harvard Medical School, Boston, MA, United States of America

5. Radiology and Nuclear Medicine, Maastricht University Medical Center+, Maastricht, The Netherlands

6. Radiation Medicine Program, Princess Margaret Cancer Centre, Toronto, Canada*.*

**^*^** Corresponding author(s): Zhenwei Shi ([zhenwei.shi@maastro.nl](mailto:zhenwei.shi@maastro.nl)).

**^†^** Zhenwei Shi and Ivan Zhovannik contributed equally.

The contents of this file are listed in the table below with page number and brief description.

|  | **Page number** | **Brief description** |
| --- | --- | --- |
| **Table A** | 3 | Radiomic feature names and formulas in the original article and PyRadiomics. |
| **Figure A** | 5 | The distribution of four features in training and testing datasets after Z-score scaling. |
| **Figure B** | 5 | The distribution of four features in training and testing datasets after Log10 data transformation and Z-score scaling. |
| **Table B** | 5 | Radiomic features distributions and split medians. |
| **Figure C** | 6 | Kaplan Meier survival curves of single radiomic feature. |

**Radiomic features**

The following four radiomic features were used in this study. Table A lists the names in the original paper and PyRadiomics. The definition and formula are described as follows.

**Table A**: **Radiomic feature names in the original article and PyRadiomics**.

|  | Hugo’s feature name | Pyradiomics name |
| --- | --- | --- |
| Feature 1 | Statistics Energy | original_firstorder_Energy |
| Feature 2 | Shape Compactness | original_shape_Sphericity ^3 |
| Feature 3 | Grey Level Nonuniformity | original_glrlm_GrayLevelNonUniformity |
| Feature 4 | wavelet Grey Level Nonuniformity HLH | wavelet-HLH_glrlm_GrayLevelNonUniformity |

**Feature 1:** **original_firstorder_Energy**


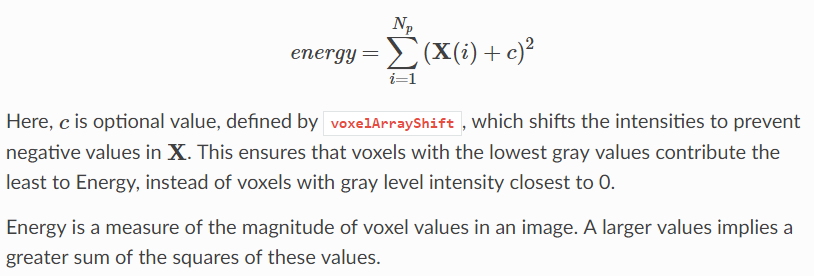


Original formula [Aerts et al]:


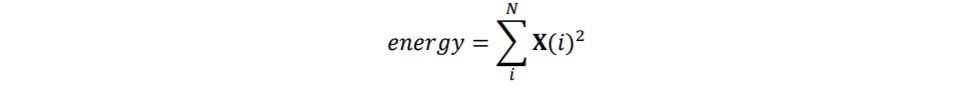


**Feature 2:** **original_shape_Sphericity**


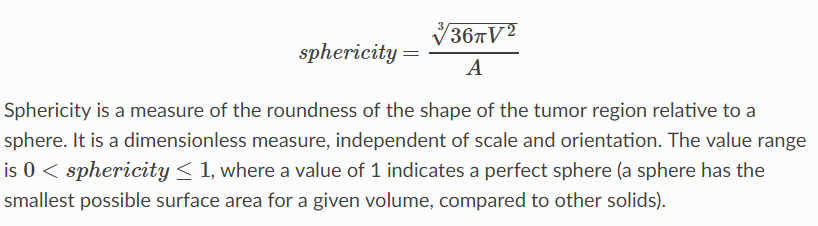


The **Compactness2** feature is highly correlated to sphericity, so the compactness2 was computed via the third power of sphericity. Original formulae [Aerts et al]:


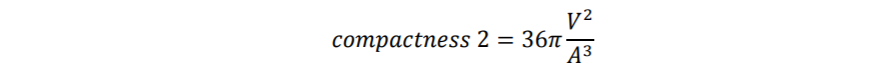


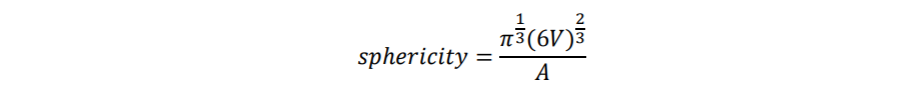


**Feature 3: original_glrlm_GrayLevelNonUniformity**


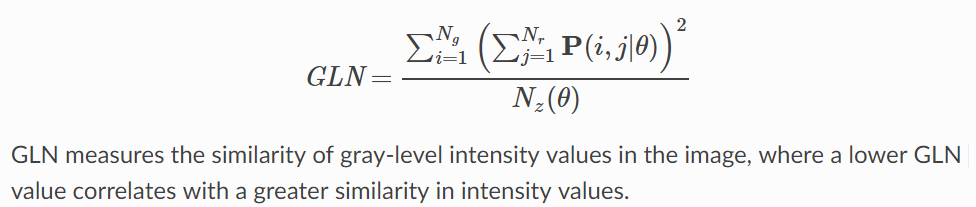


Original formula [Aerts et al]:


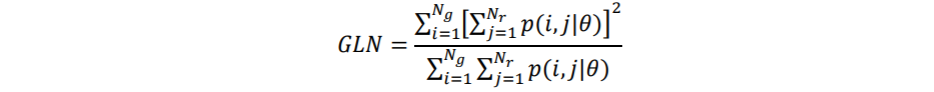


**Feature 4: wavelet-HLH_glrlm_GrayLevelNonUniformity**

The wavelet analysis was conducted using the python library PyWavelets (version 0.5.2) and ‘Coif1’ wavelet was applied as the filter on the original CT images. The formula of feature 4 is the identical to the feature 3, but on the filtered images.


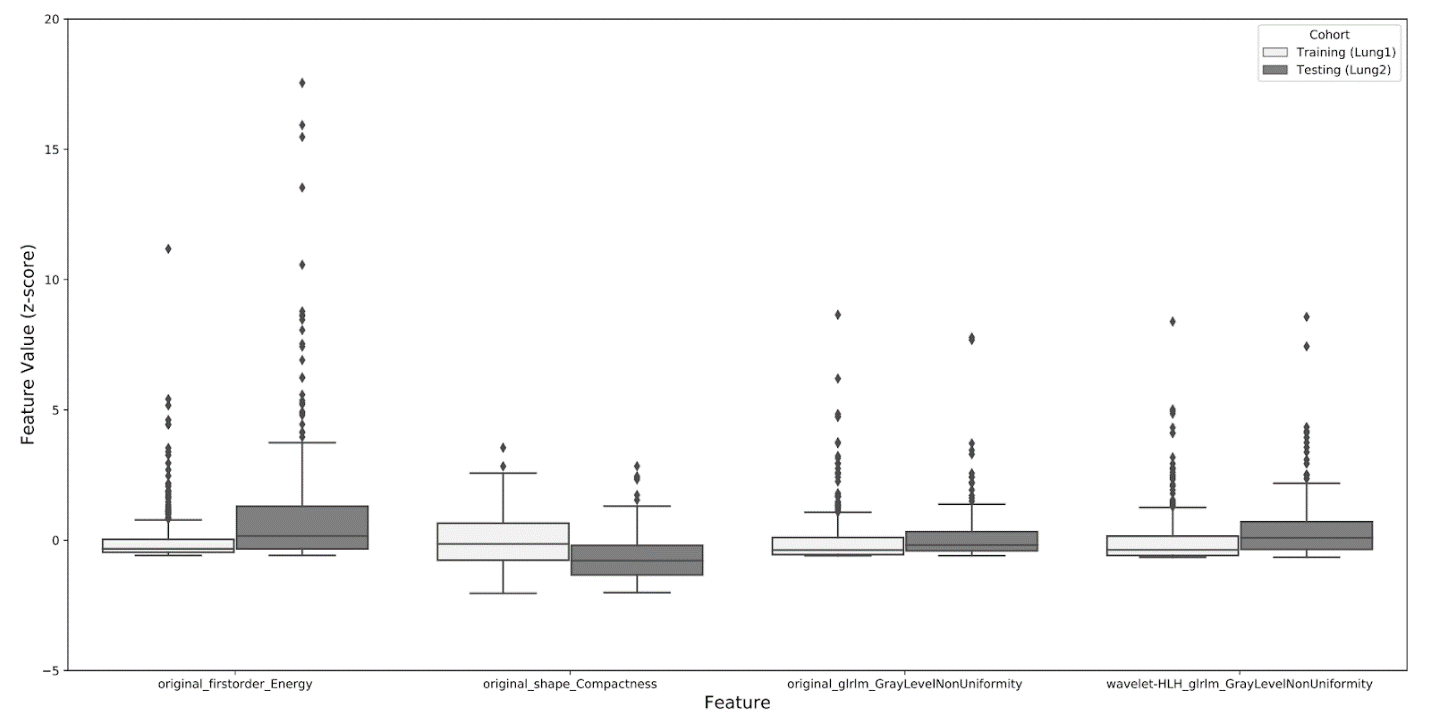


**Figure A: The distribution of four features in training and testing datasets after Z-score scaling.**


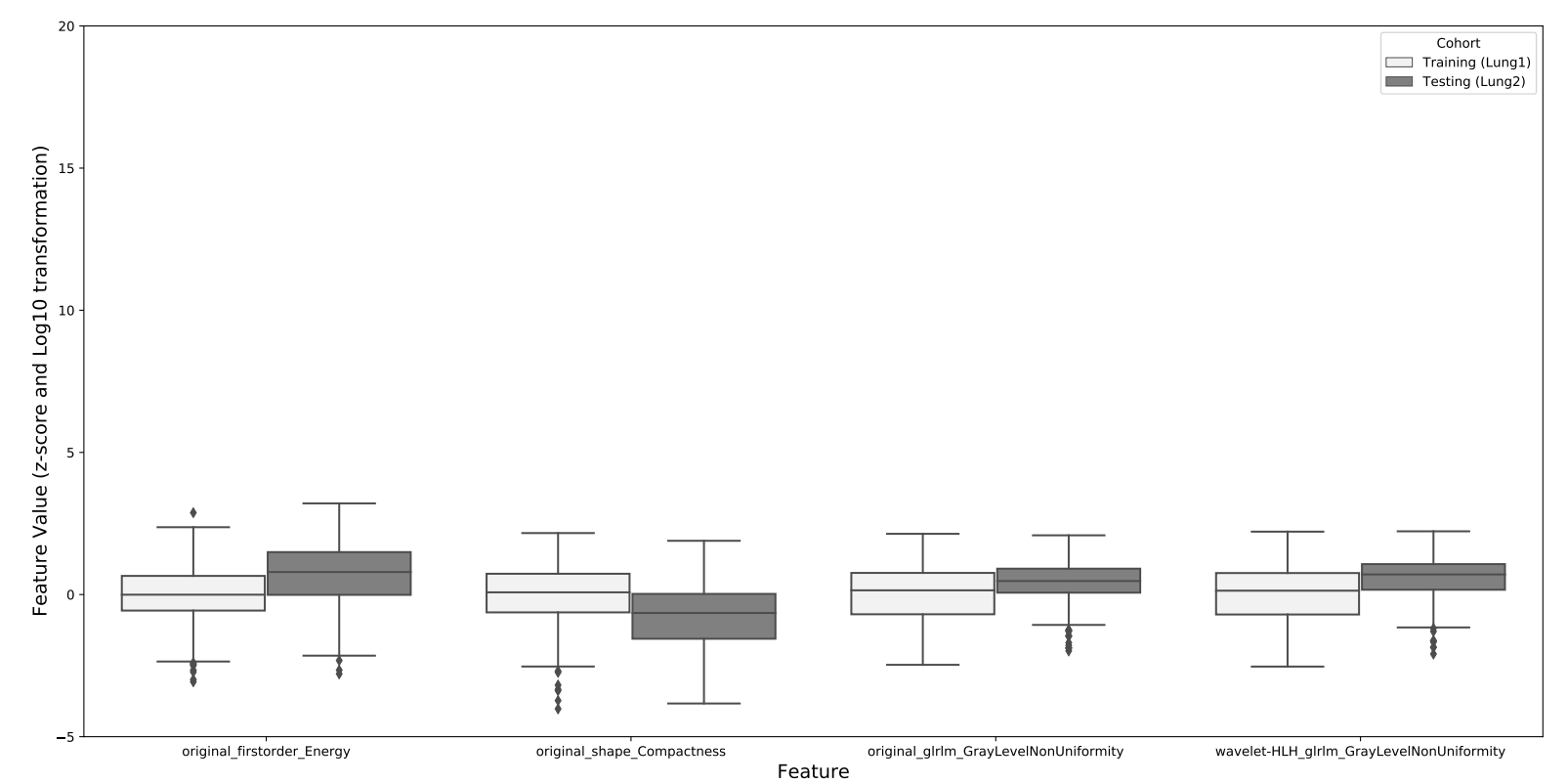


**Figure B: The distribution of four features in training and testing datasets after Log10 data transformation and Z-score scaling.**

**Table B: features distributions and split medians.**

| **Predictors** | **raw mean** | **raw std** | **scaled mean** | **scaled std** | **split median** |
| --- | --- | --- | --- | --- | --- |
| original_firstorder_Energy | 8.426e+08 | 1.431e+09 | -4.487e-16 | 1.001 | -4.915e-03 |
| original_shape_Compactness | 0.2569 | 0.1088 | 3.296e-17 | 1.001 | 7.861e-02 |
| original_glrlm_GrayLevelNonUniformity | 2542 | 4261 | -6.137e-17 | 1.001 | 1.49e-01 |
| wavelet-HLH_glrlm_GrayLevelNonUniformity | 4674 | 7103 | -6.219e-17 | 1.001 | 1.363e-01 |


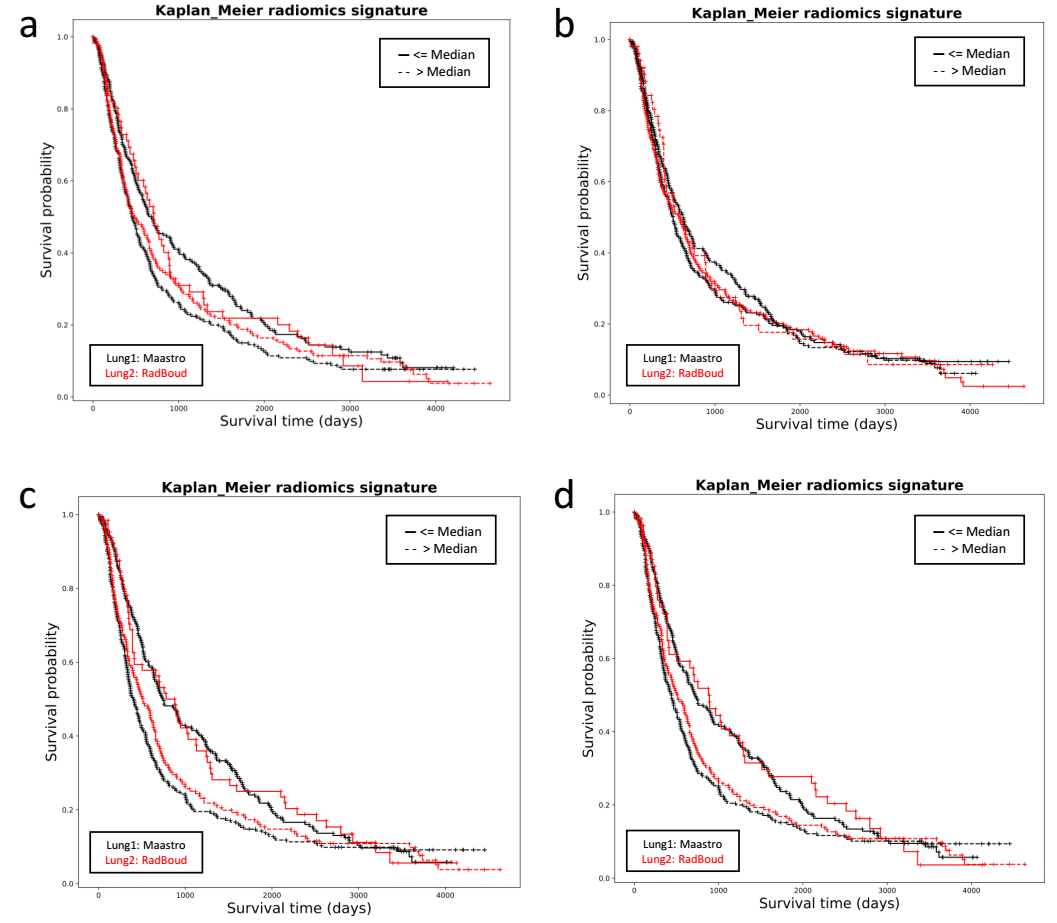


**Figure C: Kaplan Meier survival curves of single radiomic features. a original_firstorder_Energy, b original_shape_Compactness, c original_glrlm_RunLengthNonUniformity, d wavelet-HLH_glrlm_RunLengthNonUniformity.**

The statistics and split median for each of the features of the cox regression model are shown in Table B. The performance of the radiomic signature was validated in the dataset Lung2 using the Harrell concordance index (HCI), which is a generalization of the area under the ROC curve. The radiomic signature has a reasonable performance to split in the validation set (HCI = 0.58, P = 3.9 x 10^-18^, Wilcoxon test, n = 221). The Kaplan Meier curves of each single feature are shown in Figure C.
